# Role of Hemocytes in the Aging of Drosophila Male Germline

**DOI:** 10.3390/cells14040315

**Published:** 2025-02-19

**Authors:** Virginia Varga, Janka Szinyákovics, Anikó Bebes, Fanni Szikszai, Tibor Kovács

**Affiliations:** 1 Department of Genetics, Eötvös Loránd University (ELTE), H-1117 Budapest, Hungary; gina.elte@gmail.com (V.V.); janka.szinyakovics@ttk.elte.hu (J.S.); bebaniko@gmail.com (A.B.); fanniszikszai@gmail.com (F.S.); 2 Doctoral School of Biology, Institute of Biology, ELTE Eötvös Loránd University (ELTE), Pázmány Péter Sétány 1/C, H-1117 Budapest, Hungary

**Keywords:** hemocytes, germline, stem cells, JAK-STAT, aging

## Abstract

Stem cells are essential for the proper functioning of tissues, replacing damaged, senescent cells to ensure tissue regeneration. However, as age advances, the number of these stem cells can change, and their self-renewal abilities can become impaired, leading to disruption of homeostasis, loss of regenerative capacity, and, ultimately, deterioration of tissue function. In *Drosophila* testis, in addition to the germline and somatic cells involved in spermatogenesis, there are immune cells (hemocytes) with macrophage function. In our study, we aimed to investigate the role of hemocytes in maintaining germline stem cells throughout their lifespan. Our results show that in the absence of plasmatocytes and crystal immune cells, the number of germline stem cells (GSCs) and apoptotic germline cells also increases significantly during senescence, which may have detrimental effects on the differentiation processes of germline cells. The size of the hub increases in aged male testes. It is therefore conceivable that changes in the hub may induce dysfunction of differentiation processes. The fertility of aged immunodeficient animals is decreased. Furthermore, we show that the expression of the JAK/STAT signaling pathway, which is essential for the maintenance of the stem cell *niche*, is impaired in the lack of hemocytes. We found an increased expression of Socs36e, an inhibitor of JAK-STAT, which correlates with decreased JAK-STAT activity. Overexpression of Socs36e in the apical part of the germline led to a phenotype similar to the immunodeficient aged germline, where an increased GSC number and hub size were also observed. However, spermatogenesis was also disturbed in this case. Our study shows that hemocytes are required to regulate the number of GSCs. This regulation could be mediated through the JAK-STAT signaling pathway. These results may help to provide a more complex insight into the relationships between immune cells and stem cells.

## 1. Introduction

Aging is an inevitable process for human life, which causes other processes to decline or be compromised. One of these processes is tissue renewal, which could also be crucial in aging fruitfly *Drosophila melanogaster* testis. As a result of aging (or other detailed effects), the number of germ stem cells (GSCs) declines, and they need to be replaced [1]. According to the literature, the extent of GSC loss along aging is around 70–80% [2]. Due to the loss of GSCs during aging*, Drosophila melanogaster* has natural processes to replenish GSCs in the testis in response to aging or stress. The testis of the fruit fly is a suitable model to study the mechanisms of dedifferentiation and tissue regeneration during stem cell aging [3]. This model is also suitable for the investigation of the undifferentiated state of stem cells and their homeostasis [4].

The early stage of spermatogenesis in the fruitfly *Drosophila melanogaster* testis is a well-established model to study tissue senescence during aging [5]. There are two types of stem cells in the fly testis: GSCs and somatic cyst stem cells (CySCs). These two specific stem cells are physically connected with each other and a non-dividing stromal cell group, named “hub”. One daughter cell preserves its stem cell identity through distinct steps of asymmetric division of GSCs. At the same time, the other differentiates into a gonialblast (Gb), which then forms spermatogonia (SG) through a series of mitosis [6]. During the early spermatogenesis stage, germ cells undergo two cell fate changes. The first occurs during the asymmetric division of GSCs when the two daughter cells choose different cell fates. The second one takes place when SG enters into the meiotic stage (Figure S1A).

**Figure 1:**
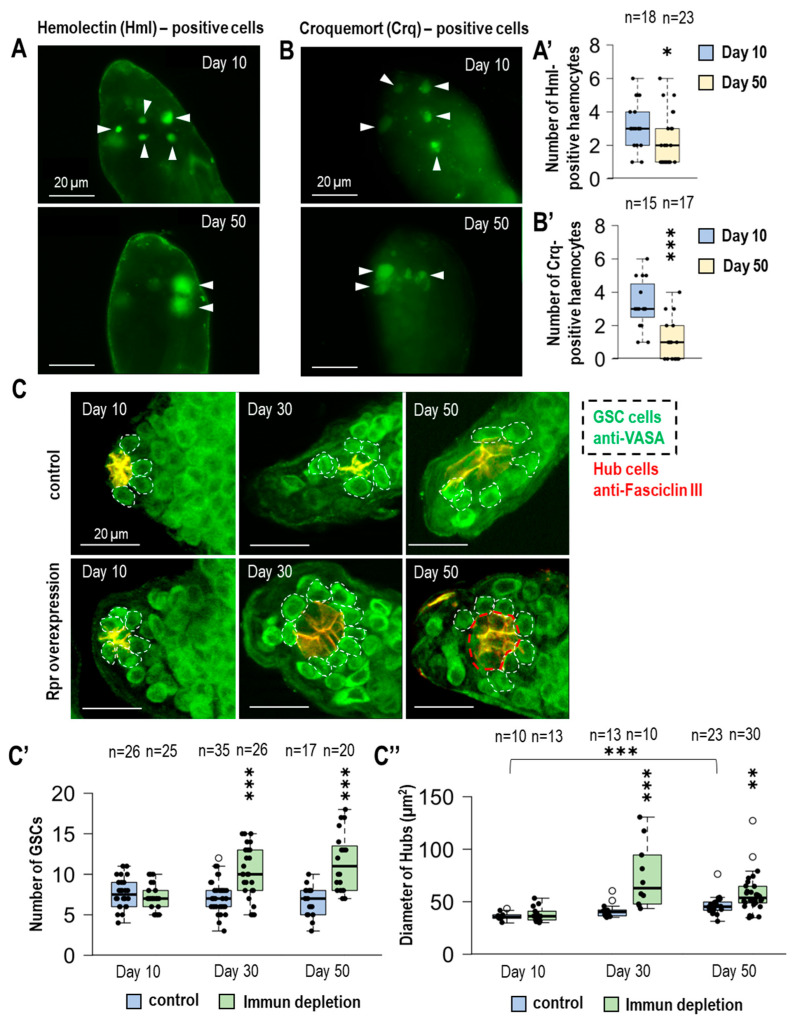
Hemocytes are necessary to regulate the number of GSCs in the male germline during lifespan. (**A**,**A′**,**B**,**B′**) Hemolectin (Hml) and croquemort (Crq) hemocyte-specific Gal4s were used to express the *UAS-GFPnls* transgene. We found a decrease in the number of hemocytes in the apical part of the germline of aged male animals. (**C**) By immunohistochemistry, we labeled the germline with anti-VASA (green) and hub cells with anti-Faciclin III (red). The VASA-positive cells adjacent to the hub were considered GSCs according to the literature (dashed line). We induced immune cell apoptosis by hemocyte-specific overexpression of *reaper* (rpr) proapoptotic factor. (**C′**) The number of GSCs was significantly increased in immunodeficient 30- and 50-day samples. (**C″**) Due to the alteration in GSC number, we also measured the change in the size of hubs in testes from control and *rpr*-overexpressing animals at various ages. We also studied the size of the hub in testes at different ages by measuring the circumference of the hubs at their largest diameter (red dashed lines). Immunodeficiency significantly increased the hub diameter in older samples compared to age-matched controls. It is worth noting that hub size also increases in control samples during lifespan. The significance level was indicated as follows: * *p* < 0.05; ** *p* < 0.01; *** *p* < 0.001.

It is important to transmit signaling ligands from the hub to the stem cells because these factors maintain stem cells in an undifferentiated state. The hub serves as a *niche* to provide an adequate microenvironment for stem cells. Hub cells supply necessary signals and cellular junctions for these cells (Figure 1). These ligands activate signaling pathways such as Janus kinase/signal transducer and activator of transcription proteins (JAK/STAT) and bone morphogenetic protein (BMP). Defects in these signaling pathways often lead to premature differentiation of stem cells [7]. JAK/STAT signaling maintains the homeostatic balance in the *Drosophila* testis. This pathway is required to maintain somatic and germ stem cell character and is activated by Upd1 and Upd3 ligands. These factors are secreted by the hub cells. Through multiple steps, Stat92E transcription factors become activated by phosphorylation, which leads to dimer formation and modifying target gene (*Socs36E, chinmo*) expression [8]. Suppressor of cytokine signaling 36E (*Socs36E*) is a negative regulator of the JAK/STAT signaling pathway, which is responsible for competition between different cell types in the microenvironment of testis stem cells. This process shows that when Socs36E is impaired, one of the cell types can overcome others, leading to an imbalance in the homeostatic state (Figure S1B) [9].

Hemocytes are members of the *Drosophila* immune system, which have similarities with some aspects of the human macrophages. During the hematopoiesis of *Drosophila*, plasmatocytes, lamellocytes, and crystal cells are formed as hemocytes. The production of plasmatocytes and crystal cells is continuous. However, lamellocytes are provided in case of immune response. The most relevant member is the plasmatocyte, which is present in many members and functions as mammalian macrophages [10]. Their other functions are diverse, such as the production of antimicrobial peptides or the induction of signaling pathways, as shown in previous studies using *Drosophila* ovaries and intestinal stem cells [11].

The main objective of our study was to investigate the role of hemocyte immune cells in the maintenance of male germline stem cell *niche* during aging. Here, we provide a paper in which we found a connection between the activity and number of hemocytes and the age-dependent change of several GSC measures in *Drosophila melanogaster* testis using the hemocytic factors introduced above.

## 2. Materials and Methods

### 2.1. Fruit Fly Stocks and Maintenance

Flies were kept at 25 °C on normal fly cornmeal nutrients [12]. Experiments were also carried out at 25 °C. Strains were ordered from Bloomington Drosophila Stock Center (BDSC). Isogenic *w [1118]* (BDSC 5905), *y [1]* w[*]; P{w[+mC]=crq-GAL4}2 (BDSC 25041), *w [1118]; P{w[+mC]=Hml-GAL4.G}6-4* (BDSC 6396), *w [1118]; P{w[+mC]=Hml-GAL4.G}6-4 P{w[+mC]=UAS-GFP::lacZ.nls}15.1 P{w[+mC]=UAS-GFP.S65T}Myo31DF[T2]* (BDSC 6397), *w [1118]; P{w[+mC]=UAS-GFP.nls}14BDSC 4775), w [1118]; P{w[+mC]=UAS-rpr.C}14* (BDSC 5824), *w [1118], P{Hml-GAL4.Δ}3, P{UAS-2xEGFP}AH3/MKRS* (BDSC 30142), *Oregon-R-C* (BDSC 5), y [1] sc[*] v [1] sev [21]; P{y[+t7.7] v[+t1.8]=TRiP.HMS01623}attP2 (BDSC 36732), w[*]; P{w[+mC]=UAS-Socs36E.R}2 (BDSC 91352), *w [1118]; 10xstat92e-GFP* [13].

### 2.2. Immunohistochemistry

Testes were pre-dissected in PBS and were prefixed with 4% formaldehyde for 45 min (dissolved in PBS) and washed three times (10 min) in PBST (Tween 20 dissolved in PBS into 0.1%). Samples were blocked for 1 h with fetal bovine serum (FBS) dissolved in PBST (1:20). Anti-VASA (1:50, rat, DSHB, 2855), anti-FasciclinIII (1:25, mouse, DSHB), anti-αTubulin (1:10, mouse, DSHB) and anti-Hts/1B1 (1:20 mouse, DSHB) were applied. Immunostaining was performed overnight at 4 °C [14]. Following primary antibody labeling, samples were washed three times for 20 min in PBST solution (the first washing was carried out with a PBST containing 5x NaCl) and blocked in FBS-PBST blocking solution at room temperature for 1 h. The following secondary antibodies were used: anti-Mouse Alexa Fluor 488 (1:500, Life Technologies Carlsbad, CA, USA, A11001), anti-Mouse Texas Red (1:500, Life Technologies Carlsbad, CA, USA, T862) and anti-Rat Alexa Fluor 488 (1:500, Life Technologies Carlsbad, CA, USA, A21210). Antibodies were diluted in a blocking solution, and and incubated for one hour at room temperature. Before covering the samples, secondary antibodies were removed by 3 PBST and 1 PBS wash (for 20 min). If nuclei staining was applied, it was conducted by Hoechst (0.1 mg/mL, Molecular Probes Eugene, OR, USA, 33342) in glycerol:PBS (4:1) cover solution.

### 2.3. Fluorescence Microscopy

Fluorescent images were captured with a Zeiss Axioimager Z2 upright microscope (with objectives Plan-NeoFluar 10x 0.3 NA, Plan-NeoFluar 40x 0.75 NA and Plan-Apochromat 63x 1.4 NA) equipped with ApoTome. ZEN 3.5 (Blue edition v3.5.093) and Image J 1.54f software were used to examine and evaluate data. The same exposure time and magnification were used for all samples during measurement.

For all fluorescence images, a Z-stack was created with a slice distance of 1 µm. For GSC numbers, we counted every new VASA-positive cell that was physically in contact with hub (FasciclinIII-positive) cells per slice. To determine the size of hub cells, we re-counted the circumference of FasciclinIII-positive cells at the largest diameter. Hub size was calculated in μm^2^. To determine the circumference, we used the Zeiss Zen Light (Blue edition v3.5.093) software.

LysoTracker Red (Life Technologies Carlsbad, CA, USA, L-7528, diluted at a ratio of 1:1000 in PBS) was used to stain acidic compartments [15]. The testes were dissected in PBS and incubated in LysoTracker Red (LTR) diluted in PBS for 1 min. After the removal of the LTR solution, the samples were washed 2x for 2 min in PBS. Nuclei were stained with 50 μg Hoechst in Glycerol/PBS (4:1) mounting solution.

### 2.4. Electron Microscopy

The testes of adult flies were dissected in ice-cold Phosphate Buffered Saline (PBS), and fixed in a solution containing 2% formaldehyde, 0.5% glutaraldehyde, 3 mM CaCl2, and 1% sucrose in 0.1 M Na cacodylate (pH 7.4) for 30 min. Postfixation was then carried out in 0.5% osmium tetroxide (45 min at room temperature) and in half-saturated aqueous uranyl acetate (30 min at room temperature). The samples were then dehydrated in a graded series of ethanol and embedded in Durcupan (Sigma-Aldrich St. Louis, MO, USA, 44610-1EA). White, according to the manufacturer’s instructions, and incubated for 36 h at 60 °C. Ultrathin sections were stained with lead citrate for five minutes [12] Samples were analyzed in a JEOL JEM 1011 transmission electron microscope operating at 60 kV. Images were captured using an Olympus Morada 11-megapixel camera and iTEM software (Olympus v5.2, Shinjuku, Japan). All reagents and materials used for electron microscopy were obtained from Sigma-Aldrich St. Louis, MO, USA.

### 2.5. Western Blot

Protein samples were prepared for Western blotting at a concentration of 1 mg/mL. Samples were collected in PBS, stored on ice, and then replaced with fly lysis solution and Laemmle buffer (1:1). Before storage, samples were homogenized and denatured at 95 °C. Protein levels were measured by Western blot on 10% SDS denaturing acrylamide gel. Used primary antibodies: anti-GFP (rat, 1:2500, Developmental Studies Hybridoma Bank Iowa City, IO, USA, DSHB-GFP-1D2), anti-Tub84B (mouse, 1:1000, Merck Life Science Darmstadt, Germany, T6199) was used as an internal control. The following secondary antibodies were used: anti-rabbit IgG alkaline phosphatase (1:1000, Merck Life Science Darmstadt Germany, A3687), anti-mouse IgG alkaline phosphatase (1:1000, Merck Life Science Darmstadt, Germany, A8438),. NBT-BCIP solution (Merck Life Science Darmstadt, Germany, 72091) (diluted in 3% milk powder TBST) was used to develop the antibody labeling [16].

### 2.6. PCR Experiments

Testes were dissected from adult males in PBS, collected in TRI Reagent^®^ solution (Zymo Research Irvine, CA, USA, R2050-1–50), and homogenized. RNA isolation was performed according to the Direct-zol™ RNA MiniPrep kit (Zymo Research, R2050) protocol, which also includes a DNAse treatment. Reverse transcription was performed using RevertAid First Strand cDNA Synthesis Kit (Thermo Fisher Scientific Waltham, MA, USA, K1621). The following primers were used in a semi-quantitative PCR analysis to amplify internal control: *rpl32:* forward—5′-GCT AAG CTG TCG CAC AAA TGG—3′; reverse—5′-GTA GCC AAT GCC TAG CTT GTT C-3′, For detection of *chinmo*: forward—5′-AGC AGT TCT GCC TCA AAT GG 3′; reverse—5′- AGA TCG GCG AAC TTC TTT GA-3′ *socs36e*: forward—5′-TCG TCG AGT ATT GCG AAG TG-3′; reverse 5′-CTG CTC CCA TTG AAA GTG CT-3′. Detection of PCR products was obtained by SYBR Green dye.

We performed Quantitative PCR with LightCycler 96 Instrument (Roche Molecular Systems Pleasanton, CA, USA) PCR. Amplification was performed using the FastStat Essential DNA Green Master kit (Roche Basel, Switzerland, 06924204011). Quantitative PCR was used with the following settings: denaturation at 95 °C for 600 s, amplification (45 cycles) at 95 °C for 10 s, 58 °C for 10 s, and 72 °C for 20 s. Melting 95 °C for 10 s, 65 °C for 60 s, 97 °C for 2 s. Cooling 40 °C for 30 s. For quantitative PCR, we used the same primers as described above.

### 2.7. TUNEL

Fixing adult testis was conducted according to Immunohistochemistry explained above. TUNEL (Terminal deoxynucleotidyl transferase dUTP nick end-labeling) assay was performed with the following reagents: Equilibrium Buffer (Merck Millipore Burlington, VA, USA, S7106), Reaction Buffer (Merck Millipore Burlington, VA, USA, S7105), TdT enzyme (Merck Millipore Burlington, VA, USA, S7107) anti-digoxigenin-AP (Roche Basel, Switzerland, 11,093,274,910), NBT-BCIP solution (Sigma-Aldrich St. Louis, MO, USA, 72,091) [16,17]. For TUNEL-positive structures, we considered 3.15–7 µm^2^ markers to be derived from somatic cells, while apoptotic structures between 15.5–31.5 µm^2^ were defined as germline cells. The two TUNEL-positive markings, separated by size, were plotted separately and examined for significance from a statistical perspective.

### 2.8. Evaluation, Statistics

Results were determined using R Studio for statistical fluorescence microscopy analysis (Version 3.4.3). The distribution of samples (to decide normality) was tested with Lilliefor’s test. If it was normal, an F-test was performed to compare variances. In cases when variances were equal, for clonal results, a two-sample *t*-test was used. Otherwise, a one-sample *t*-test was applied. In the case of non-normal distribution, the Mann–Whitney U-test was performed. The significance level was indicated as follows: * *p* < 0.05; ** *p* < 0.01; *** *p* < 0.001.

## 3. Results

### 3.1. The Number of Immune Cells Located in the Testes Varies During Aging

Identifying the number of hemocytes present in the testis would help to understand if there is a link between the number of immune cells and the balance change of the stem cell *niche* in the testis. Studies have revealed new molecular markers to identify them, such as Hemolectin (Hml), which is specific to plasmatocytes and crystal cells [18]. Hemolectin protein consists of multiple subunits, some of which are conserved and can also be found in human vWF (von Willebrand factor), which is a hemostatic factor and also crucial in coagulation and complementation factors [19,20]. Another important factor in identifying hemocytes is a *Drosophila* homolog of the vertebrate CD36 scavenger receptors called Croquemort (Crq). The primary function of this receptor is to establish the proper immune response through phagocytosis of pathogens. We also observed the phagocytic function of hemocytes in testis using fluorescence and electron microscopy (Figure S2A). In addition to the cellular immune response, *Drosophila melanogaster* also has a humoral immune response. Using Hemolectin (*HmlΔ* promoter drove Gal4 here after called *Hml-Gal4*) allowed us to find a correlation in the frequency of occurrence. This protein is expressed only in fully differentiated hemocytes and restricted to the subpopulation of both plasmatocytes and crystal cells; however, it is never found in lamellocytes [19]. To obtain the number of hemocytes, we triggered *UAS-GFPnls* (GFP with a nuclear localisation signal) expression with Hml-Gal4. As a result, it was detectable that in 50-day-old adults, the number of plasmatocytes and crystal cells decreased (Figure 1A,A′). It was also in our interest to define whether the number of hemocytes decreases with aging using Crq-Gal4 driver with Hml-Gal4 detection. In addition to its role in phagocytosis, Crq also contributes to direct and indirect proper immune response during the pathogen invasion [18,21]. The number of Crq-positive hemocytes is also significantly reduced in 50-day-old individuals. Examining both hemocyte markers revealed a significant decrease in immune cell number (Figure 1B,B′).

### 3.2. Immunodeficiency Results in an Increasing Number of GSCs in Aging Fruit Flies

During aging, the number of hemocytes decreases significantly, and in our next experiment, we investigated the role of hemocytes on the change in GSCs during aging. It has already proven that continuous sperm production needs the homeostatic function of the stem cell *niche*, which can be found in the apical site of the testis. Aging and other molecular events and signaling pathways, along with this process, can affect the balance of the *niche*, which can modulate the number of GSCs and lead to insufficient sperm production [1]. It has already been shown that the induction of immune depletion using the pro-apoptotic factor *reaper* (*rpr*) causes inadequate stem cell *niche* operation in the ovaries of female *Drosophila* flies [22]. In male flies, to further investigate the consequences of hemocyte depletion in the specific case of GSC number, we conducted experiments using a Gal4 system driven by the Hml-Gal4 to induce *reaper* overexpression. The success of immune depletion was confirmed by a significant reduction in Hml-positive GFPnls cells in testes by microscopy and Western blot methods (Figure S2B,C). Subsequently, immunohistochemistry was used to detect hub cells (anti-FasciclinIII) and germline cells (anti-VASA) in test samples of F1 males (10, 30, and 50 days old) obtained from the crosses. The results show that the number of germline cells in control animals gradually decreases, and the number of GSC cells increases with age in the case of *rpr* overexpression. Interestingly, while in the absence of hemocytes, the number of GSCs significantly increases in 50-day-old testes under immunodeficient conditions compared to controls (Figure 1C,C′). Taken together, it can be concluded that in the absence of immune cells, the number of germline stem cells increases significantly in aged males. Thus, during aging, the reduction in the number of hemocytes may impair certain regulatory processes, disrupting the homeostatic function of the stem cell *niche*.

### 3.3. The Size of the Hub Is Changing During Aging

Changes in hub size can trigger both an increase in the number of GSCs and a disruption of the *niche*’s homeostasis. A trigger of hub enlargement can be a malfunction in differentiation. This process occurs due to the disruption of cell junctions (the occluding junction that forms a permeability barrier during regular differentiation events in testes). Somatic cyst cells can be transformed into hub cells [23].

**Figure 2:**
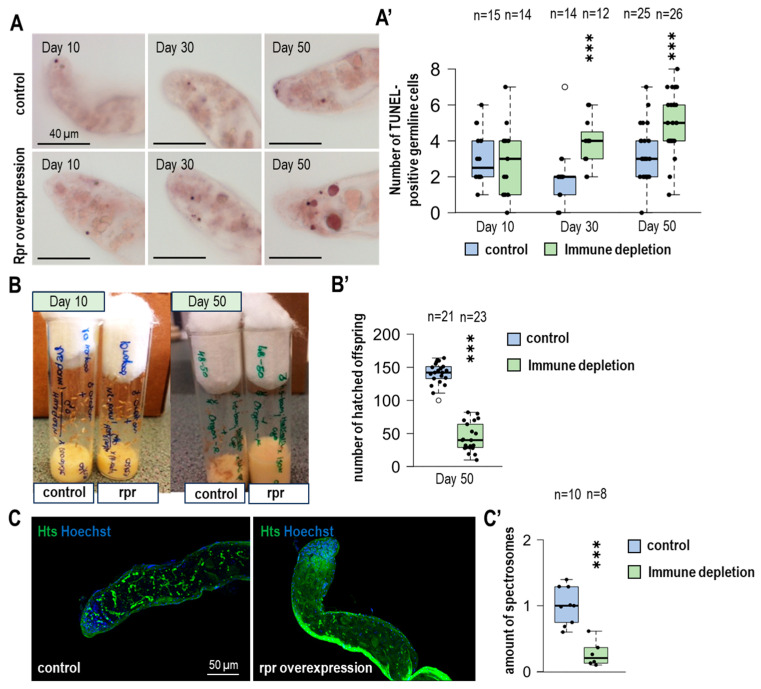
*Drosophila* male fertility declines during the lifespan due to the absence of immune cells. (**A**) Apoptotic cells were labeled with TUNEL assay in control and immunodeficient testes of different ages. (**A′**) Germline cells have larger nuclei. These TUNEL-positive cells are quantified. Our results show increased apoptosis at the apical tip of old immunodeficient male germlines. (**B**,**B′**) We compared the fertility of control and immunodeficient young and old males. Males were crossed one at a time with wild-type Oregon virgin females. In young animals, no differences were found between the two investigated genotypes. However, at age 50 days, the fertility of immunodeficient males was reduced, with significantly fewer offspring than age-matched controls. (**C**) Anti-Hts/1B1 labeling (green) was used to examine the number of fusome structures in the testes of 50-day-old control and immune-depleted animals. (**C′**) Fewer fusomes were observed in immune-depleted testes. We used Hoechst for nuclei staining. The significance level was indicated as follows: *** *p* < 0.001.

In the case of immune depletion during aging, an increase in the number of GSCs could be observed, which led to examining the link between increased GSC number and the changes in the size of the hub under immune-depleted conditions. The size of the hub increased progressively in *rpr* overexpressing animals (Figure 2C,C′). The lack of immune cells could be due to increased hub size under immune-depleted conditions. The hub is significantly enlarged during aging, which can contribute to the disruption of the germ line and cystic stem cells connected to it [24]. The aged testis showed a small but significant increase in hub size, which can be explained by reduced endogenous hemocyte numbers. Immune depletion induced an earlier effect. In 30-day-old animals, hub size increased only in the case of Hml-Gal4 driven *rpr*.

### 3.4. The Number of Apoptotic GSCs Is Increasing Due to the Lack of Hemocytes

Hemocytes, which reside in the apical part of the testis, are capable of phagocytosis, and their presence has been demonstrated by microscopic techniques. These cells also have the ability to eliminate the already dysfunctional spermatogonial cysts by forming appendages around them.

To examine the changes in the number of apoptotic germ cells under immune-depleted circumstances during aging, we conducted TUNEL assay. This method stained the fragmented DNA, allowing the identification of apoptotic germline cells [25]. For the apoptosis analysis, large germline and small somatic TUNEL-positive nuclei were counted separately in the apical region of the testes (Figure 2A,A′ and Figure S3A).

**Figure 3:**
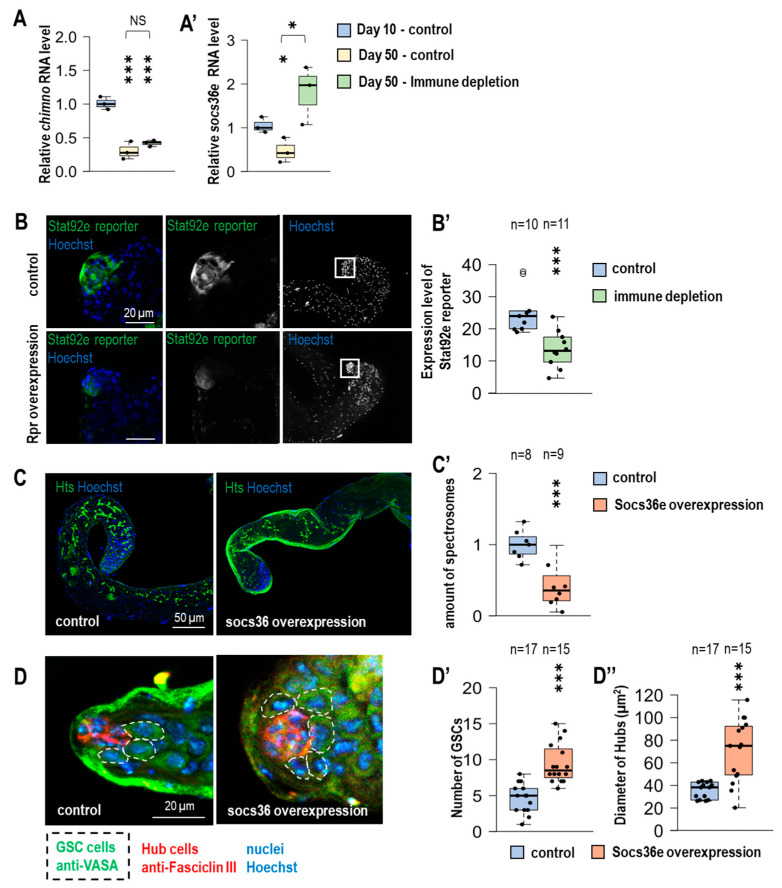
The lack of immune cells leads to increased expression of socs36e and inhibition of JAK-STAT. (**A**,**A′**) Quantitative real-time PCR was used to investigate the mRNA levels of two well-known target genes of JAK-STAT (chinmo and Scocs36e) in young (10 days old) and old (50 days old) control and immune cell-deficient animals. The mRNA was isolated from the testes of the animals. Immune depletion was induced by overexpression of *rpr* in Hml-Gal4 positive cells. Both *chinmo* and *socs36e* expression were downregulated in aged control animals. However, increased *socs36e* mRNA levels were detected in 50-day immunodeficient animals. (**B**) The activity of the JAK-STAT signaling pathway was investigated by using 10xStat92e-GFP (green) reporter. Stat92e is a transcription factor of the JAK-STAT signaling pathway, and the expression of the regulated transgene can monitor its activity. (**B′**) In immunodeficient cells, the expression of 10xStat92e-GFP reporter was significantly decreased. (**C**) Anti-Hts/1B1 labeling (green) was used to examine the amount of fusosome structures in the testes in 50-day-old animals. Nanos-Gal4 was used to induce Socs36e overexpression in germline stem cells (GSCs) and gonialblast cells. (**C′**) Elevated expression of Socs36e reduced the amount of fusosome structures. (**D**) We examined the effect of Socs36e overexpression on both the number of GSCs and the size of the hub. Similar results were obtained in immunodeficient animals, (**D′**) significant increase in GSC number and (**D″**) hub size was observed. Anti-Fasciclin III (red) was used to label hub, and anti-VASA (green) antibody labels were used to label germline cells. In fluorescence images, nuclei were labeled using Hoechst staining. The significance level was indicated as follows: * *p* < 0.05; *** *p* < 0.001.

During aging, the number of germ cells that will die significantly increases due to the lack of immune cells (Figure 3A′). Strikingly, we found no difference in the number of small somatic TUNEL-positive cells in immunodeficient and control samples (Figure S3A). Based on these observations, it can be hypothesized that the increasing number of apoptotic germ cells can arise due to the lack of phagocytotic cells in older testes.

### 3.5. In the Lack of Immune Cells, Fertility Is Decreasing During Aging

A decreased frequency of occurrence and increased number of stem cells and hub size can be observed during aging. At first sight, the increased number of GSCs can be linked to increased fertility due to sperm-producing cells. However, there is more to it; since the increasing number of GSCs causes an imbalance of the homeostatic operation of stem cell *niche* along with the impairment of the regulatory mechanisms of the differentiation process, the correct steps in the sperm production process are not activated, resulting in reduced fertility in individuals.

Due to the arising changes present under immune-depleted circumstances, we conducted fertility tests. In these tests, one male (*UAS-rpr* and control) was crossed with two females (Oregon-R) in vials. After two days, we discarded the individuals from the tubes. Then we examined the progeny (pupae and adults) present on the inside of the tube wall after five days (Figure 2B,B′ and Figure S3B).

The 10-day-old individuals show a minimally changed fertility, but no significant change exists between *UAS-rpr* and control fertility tests. However, when the pupae or adults from the 50-day-old animals were examined, more than half of the vials that lacked hemocytes showed reduced fertility compared to control vials. After discarding the individuals from the tubes, there was a remarkable change in the average number of hatched offspring (Figure 2B′). We examined the apical part of the testis for division anomalies. Anti-tubulin was used to label mitotic spindles and Hoechst to label chromosomes. Examination of fixed testes did not reveal differences in mitotic index or metaphase spindle morphology (Figure S3C,C′). During meiotic division, spermatogonia (SG) develops in cysts. The connection between SGs is provided by ring canals and fusomes. Fusomes were examined using anti-Hts/1B1 immunolabelling (Figure 2C). In immunodeficient testis, less anti-Hts labeling was observed, indicating inadequate development of SG cells (Figure 2C′). After opening the vesicula seminalis, we examined the movement of the mature sperm. No differences were found between the movements of sperm of aged control and immune-depleted animals. For sperm count determination, Hoechst nuclear stain was used. We found that significantly fewer mature sperm can be found in the vesicula seminalis of immunodeficient males (Figure S3D,D′).

Taken together, these findings suggest that aging under immune-depleted conditions reduces the fertility of individuals compared to controls, due to the disruption of several immune cell signaling pathways that can affect the division and differentiation of germ stem cells.

### 3.6. Expression of JAK-STAT Target Genes Decreases Under Immune-Depleted Circumstances in the Testes of Advanced-Aged Fruit Flies

The JAK-STAT signaling pathway plays a critical role in the homeostatic processes of the testes. The target genes of this novel signaling pathway consist of *chinmo,* which represses different genes involved in the differentiation process and also promotes protein degradation through post-translational modification of the protein degradational process, which contributes to the self-renewal processes of CySCs [26]. The protein, which originates from the *socs36e* gene, regulates the EGFR/MAPK signaling pathway, promoting the differentiation of CySC cells and maintaining the homeostatic operation of the stem cell *niche* [27]. Furthermore, Socs36e inhibits the JAK-STAT signaling pathway by negative feedback [9].

We examined the above-mentioned target genes of JAK-STAT signaling pathway by conducting qPCR to identify their expression level and its changes during aging, in 50-day-old males, under immune-depleted circumstances (Figure 3A,A′).

*Socs63e and chinmo* mRNAs were significantly decreased in germline samples isolated from 50-day-old animals compared to 10-day-old animals. Immunodeficiency did not alter the expression of *chinmo* in aged animals, whereas the expression of *Socs36e* was significantly increased compared to age-matched controls (Figure 3A,A′). Stat92e is a transcription factor in the JAK-STAT signaling pathway, and its activity was assessed by changes in the expression of the 10xStat92e-GFP reporter [13]. Significantly lower 10xStat92e-GFP reporter expression was observed in immunodeficient 50-day-old testis compared to age-matched controls (Figure 3B,B′). Our results show that JAK-STAT activity is reduced in the immunodeficient aged germline. Finally, we examined whether the observed changes were associated with increased levels of Socs36e (Figure S4A). Using Nanos-Gal4, we induced Socs36e overexpression exclusively in stem cells and gonialblasts [14]. Overexpression of socs36e reduces the amount of Hts-positive fusomes (Figure 3C,C′) and mature sperm (Figure 3B,B′). It also increases the GSCs and the hub size in 50-day-old animals (Figure 3D,D′). Our results suggest that immune cells are involved in the regulation of early spermatogenesis. In the absence of hemocytes, expression of Socs36e is enhanced, leading to increased hub size and stem cell number. Despite the increased number of GSCs, differentiation defects in spermatogenesis can be observed. These resulted in decreased fertility and lower sperm numbers.

## 4. Discussion

The results presented in this study suggest that the number of hemocytes, the immune cells that reside in the *Drosophila* testes, varies at different ages of the fruit fly. This was demonstrated by using different molecular markers specific for certain types of immune cells, which showed a significant decrease in the number of hemocytes in aged testes (50 days old) in the case of both Crq- and Hml-Gal4 drivers. Moreover, we also investigated the effect of the decrease in hemocytes during aging on the development of GSCs. We also revealed that the changes in the number of GSCs under immune-depleted conditions by overexpressing the pro-apoptotic factor, *reaper* (*rpr*), resulted in an increasing number of GSCs associated with aging. It is worth emphasizing that the overproduction of *rpr* with Hml-Gal4 did not affect the number of lamellocytes. It may be worth investigating in the future whether lamellocytes are present in the male germline of *Drosophila*. If present, how does their abundance vary, and do they play a role in regulating germline stem cell number and hub size? In the search for the causes of the elevated number of GSCs, we observed that the size of the hub led to an increase. Hub size was progressively increased in *rpr* overexpressing animals during aging. In the following steps, we examined how fertility is affected by the above changes. By conducting a fertility test, we also demonstrated that the aged, immunodeficient fruit flies showed a decreased number of sperm and also decreased fertility. We further investigated the possible role of resident hemocytes in the testes by performing immune labeling of fusomes and TUNEL assays, which revealed a significantly decreased fusome abundance and increased number of dying germ cells in immunodeficient and aging individuals, showing that there is another possibility, given our earlier experiments, that there is an impairment in germ stem cell differentiation and division processes, resulting in increased apoptotic cells, which may lead to reduced fertility. There is another possibility, based on our experiments, that impairment is arising in germ stem cell differentiation and division processes, which results in increased apoptotic cells and can lead to decreased fertility [28,29]. 

We also show how the expression levels of target proteins in the JAK/STAT pathway can change in the testes of aging fruit flies without immune cells. Previous findings in the regeneration of the fruit fly intestine further support this model. The function of the hemocytes in regeneration is already shown in the renewal of intestinal stem cells as a response to injury by secreting signaling pathway ligands. Plasmatocytes can secrete Upd3, a ligand of the JAK/STAT signaling pathway, that can initiate transcriptional activity of genes that are important for generating accurate immune responses to the type of infection. These data show that the Crq receptor has an indirect role in generating a humoral immune response through its ability to induce phagocytosis [18]. At the onset of pathogenic infection, intestinal hemocytes produce Dpp ligands, which induce their proliferation [30,31]. Hemocytes may also be important in the GSC *niche* in the ovary. It is shown that hemocytes produce factors required for basement membrane formation around GSCs and, through the production of ColIV components, establish a close interaction between GSCs and their microenvironment. They also help to direct the ligands of the Dpp/BMP signaling pathway to the apical part of the ovary. In the absence of hemocytes, there is also an increase in the number of germline stem cells in the ovaries [22]. Thus, the role of immune cells in regulating GSC numbers in both sexes is observed in *Drosophila*.

The aberrant division of CySC cells may increase the size of the hub [23], which may indirectly result in the result we observed: a significant increase in the number of GSCs in immunodeficient animals. In the lack of hemocytes, the JAK/STAT signaling pathway may be dysregulated, resulting in decreased expression of *chinmo* and *socs36e*; this phenomenon may impair the renewal processes of CySCs, leading to impaired function of the testis *niche* [32]. The increased expression of *socs36e* in the immunodeficient individuals is probably due to the elevated size of the hub. Overexpression of socs36e in stem cells also caused an increase in hub size and GSC number (Figure 3D–D″). We saw, in this case, a phenotype similar to the immunodeficient condition, with reduced fusome abundance and sperm number (Figure 3C and Figure S4B,B′). The effect of Socs36e, which is involved in negative regulation and termination of the JAK/STAT pathway [33], possibly leads to compromised GSC identity. Based on the literature, hub size growth is also regulated by Notch and EGFR signaling pathways [24]. Further studies may also be worthwhile in investigating the role of these signaling pathways in regulating the hemocyte stem cell population.

## 5. Conclusions

Taken together, our results and data identified novel links between aging, stem cell identity and immune cells in the *Drosophila melanogaster* testis. Here, we also propose that the role of hemocytes during aging is strongly connected to the Socs36e expression and activity of the JAK/STAT signaling pathway.

## Data Availability

The datasets used and/or analyzed during the current study available from the corresponding author on reasonable request.

## Supplementary Materials

The following supporting information can be downloaded at: https://www.mdpi.com/article/10.3390/cells14040315/s1, Figure S1: Early spermatogenesis and germline stem cell regulation in Drosophila; Figure S2: Overexpression of rpr (with Hml-Gal4) induced extensive hemocyte loss; Figure S3: Immunodepletion does not increase somatic cell death during the lifespan; Figure S4: Overexpression of socs36e may contribute to reduced sperm count.
