# Supplementary material for: Role of Hemocytes in the Aging of Drosophila Male Germline
Source: Cells. 2025 Feb 19;14(4):315. doi: 10.3390/cells14040315 (PMC11854897; doi:10.3390/cells14040315)
Supplement: Supplementary file 1 [file cells-14-00315-s001.zip › cells-3410127-supplementary.pdf]

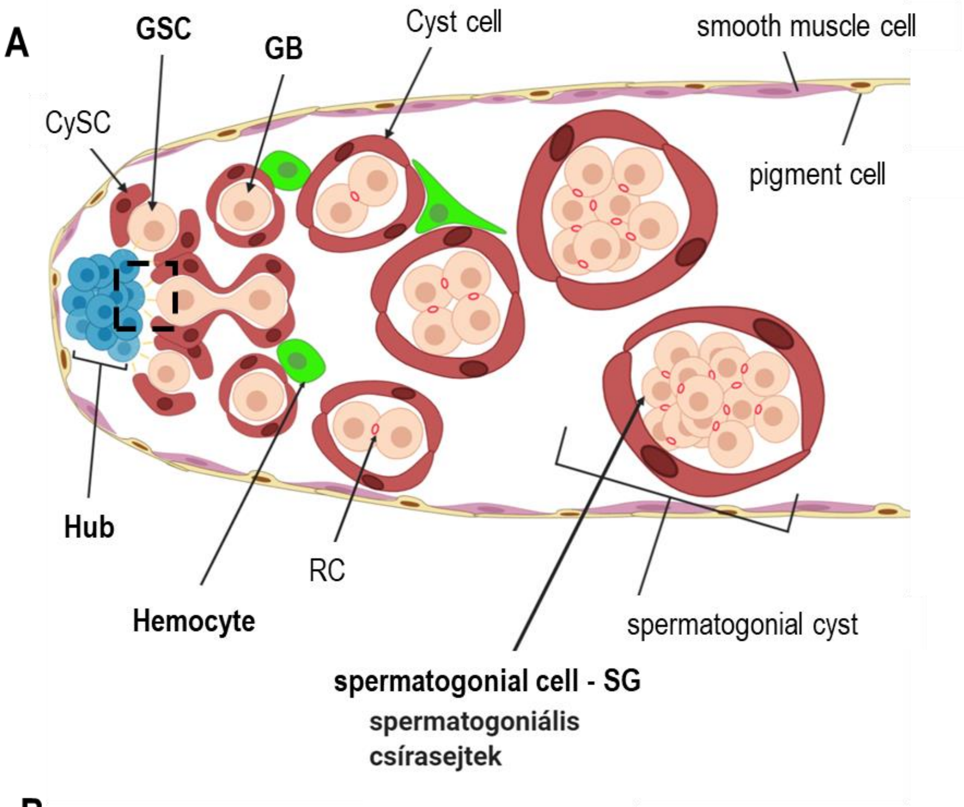

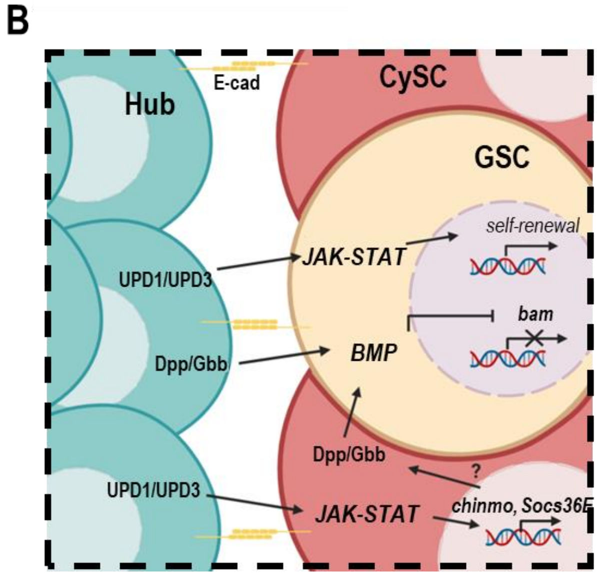

Figure S1. Early spermatogenesis and germline stem cell regulation in Drosophila. A) The apical region of the Drosophila testis contains diploid somatic and germline cells. The Hub is a cell cluster of non-dividing somatic cells that designate the location of germline stem cells (GSCs) and somatic/cyst stem cells (CySCs). GSCs produce 2 progeny cells by asymmetric division. One of them maintains its GSC function, while the other one starts differentiation after leaving the stem cell niche. This cell is called a gonialblast (GB). The GB cells are surrounded by 2 cyst cells, forming the cyst. In the cyst, the GB cells produce 16 spermatogonial (SG) cells with 4 mitoses. During divisions, the germline cells are synchronized by large ring canal (RC). Early spermatogenesis also involves immune cells, hemocytes, which have a function similar to mammalian macrophages. **B)** The undifferentiated state of GSC and CyCS cells is maintained by signaling pathways and cell junctional structures (e.g. E-cad) from the Hub. Among the signal transduction pathways of the stem cells niche, the JAK-STAT and BMP signaling pathways are the most important. The UPD1/UPD3 ligands (expressed by the Hub) activate the

JAK-STAT pathway in GSC and CyCS cells. On the other hand, the Dpp/Gbb ligands of BMP signaling activate BMP in GSCs from the Hub and CySC cells.

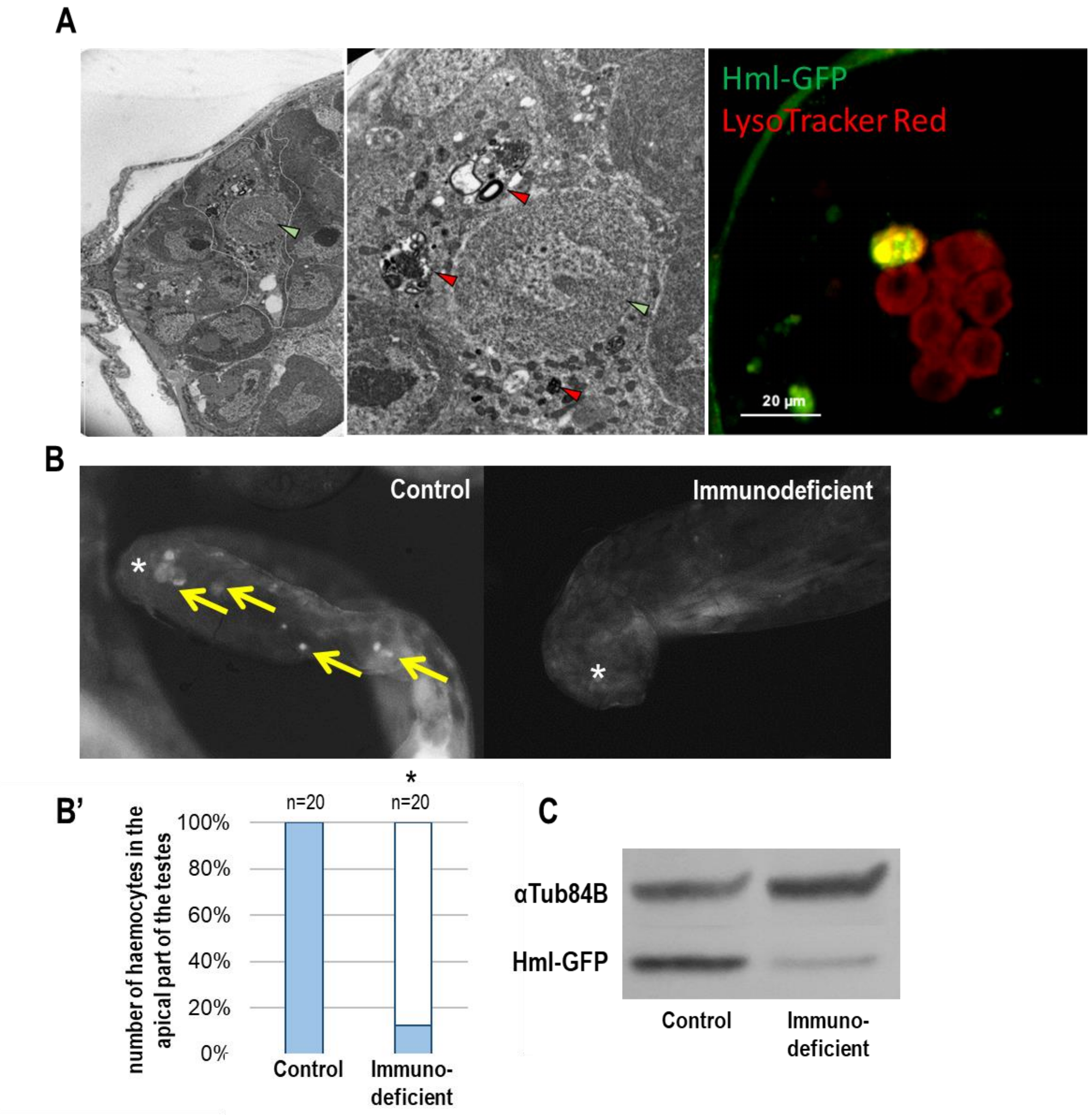

Figure S2: Overexpression of rpr (with Hml-Gal4) induced extensive hemocyte loss. A) The electron microscope image shows a hemocyte inside the testis under the epithelial cell layer. Red arrowheads indicate degrading structures (autolysosomes), while a phagocytosed germline cell is marked with a green arrowhead. Hemocytes were visualized with GFP-nls driven by Hml-Gal4. The image shows LysoTracker Red-positive spermatogonia in red. Both electron microscopy and fluorescence images show that immune cells can phagocytose germline cells in early spermatogenesis. **B-B')** Immunodepletion significantly reduced the number of GFP-positive immune cells in testis in rpr-overexpressing animals. Yellow arrows indicate hemocytes (with nls-GFP) and white asterisks indicate the apical tip of testis. **C)** Protein samples isolated from testis also showed a large decrease in hemicytic GFP labeling. Anti-αTub84B labeling was used as an internal control for western blotting.

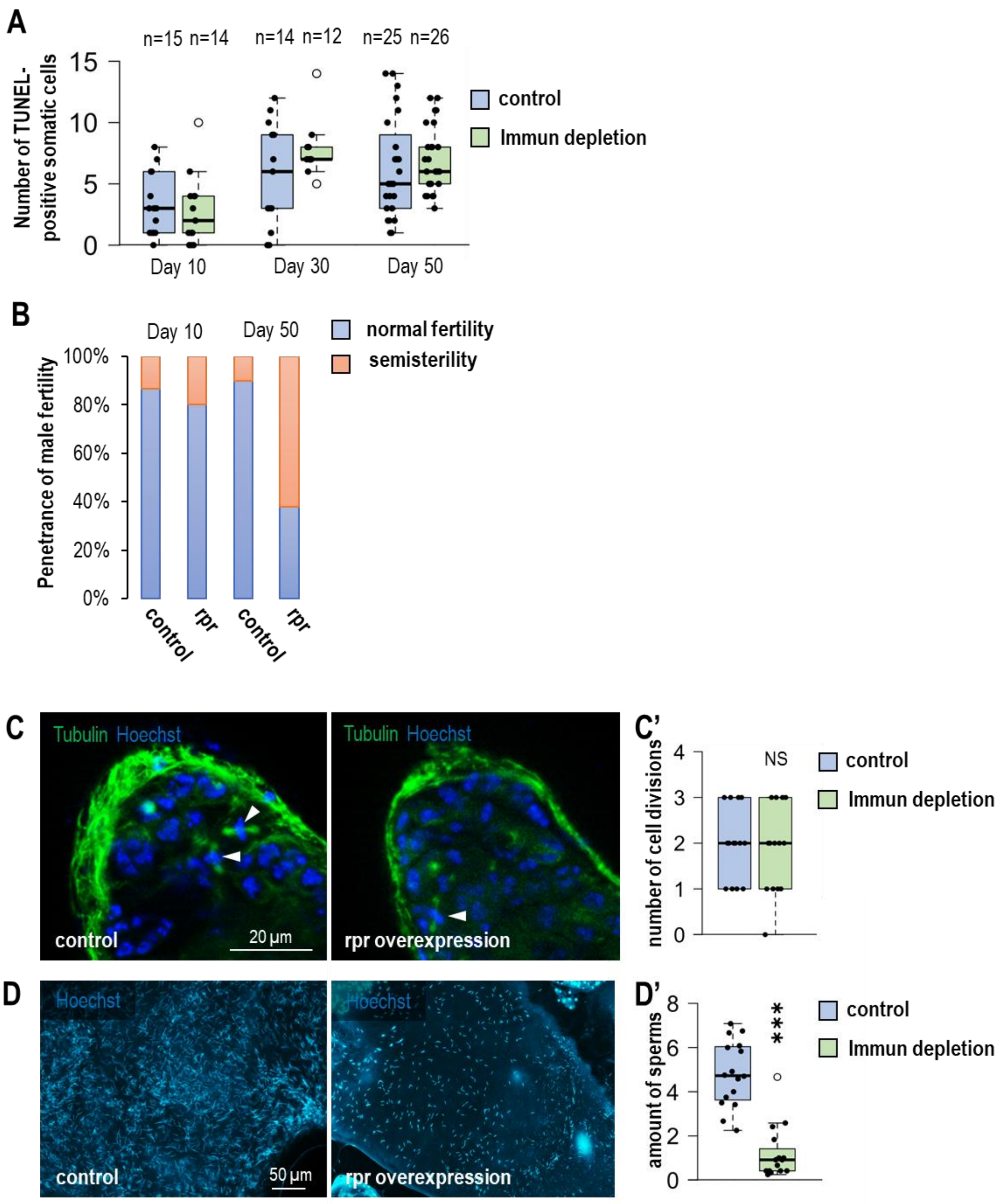

Figure S3: Immunodepletion does not increase somatic cell death during the lifespan. A) TUNEL labeling was used to measure the amount of apoptotic cells in somatic (small cell nucleated) cells throughout the lifespan. We compared TUNEL-positive nuclei in normal and immunodepleted testes in different aged samples. No differences were found between the two genotypes at either age tested. **B)** We compared the fertility of control and immunodepleted young and old males. Males were crossed one by one with wild-type Oregon virgin females. In young animals, no differences were found between the two investigated genotypes. However, at age 50 days, the fertility of immunodeficient males was reduced. The diagram

shows the penetrance of fertility of control and immunodepleted males at different ages. **C-C')** We examined differences in apical cell division of testis from 50-day-old control and immune cell-deficient (UAS-rpr overexpressing Hml-Gal4) animals. Dividing cells were labeled with anti-αTubulin and Hoechst nuclear stain. **D-D')** We compared the number of mature sperms in the seminal vesicles of 50-day-old animals in the control and Hlm-Gal4-driven per overexpressing animals. The DNA content of spermatozoa was marked with Hoechst

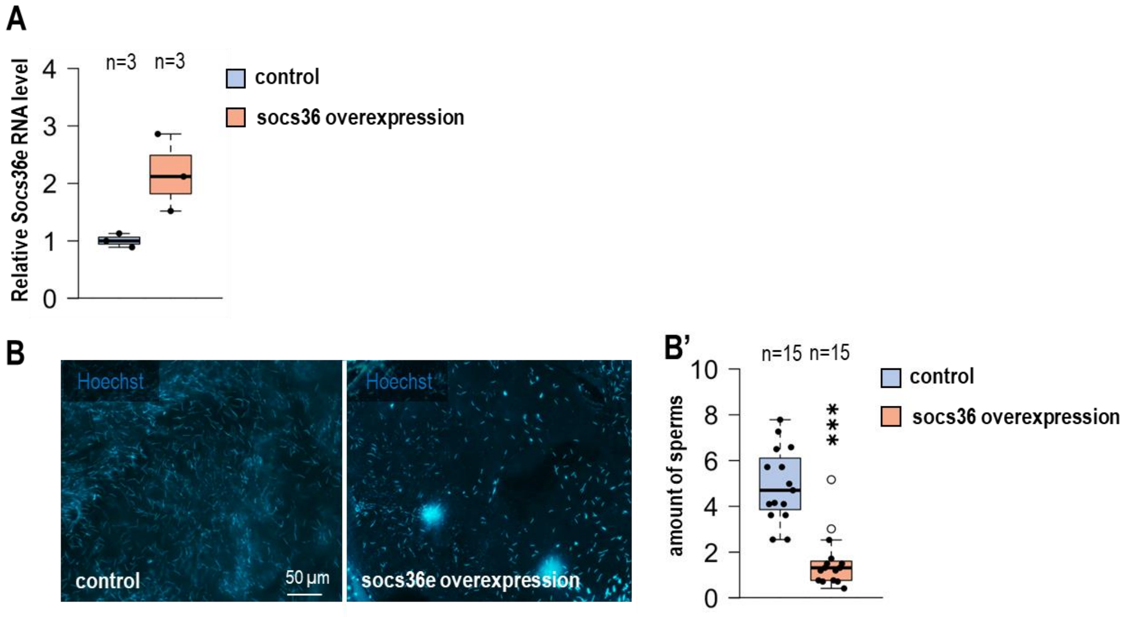

Figure S4: Overexpression of socs36e may contribute to reduced sperm count. A) We demonstrated by quantitative real-time PCR that UAS-soscs36 can induce soscs36 overexpression in Drosophila male germline. **B-B')** Overexpression of socs36e reduces the amount of mature sperms.
